# Efferocytosis Mediated Modulation of Injury after Neonatal Brain Hypoxia-Ischemia

**DOI:** 10.3390/cells10051025

**Published:** 2021-04-27

**Authors:** Jana Krystofova Mike, Donna Marie Ferriero

**Affiliations:** 1 Department of Pediatrics, University of California San Francisco, San Francisco, CA 94143, USA; Donna.Ferriero@ucsf.edu; 2 Department of Neurology Weill Institute for Neurosciences, University of California San Francisco, San Francisco, CA 94143, USA

**Keywords:** efferocytosis, stroke, neonatal brain

## Abstract

Neonatal brain hypoxia-ischemia (HI) is a leading cause of morbidity and long-term disabilities in children. While we have made significant progress in describing HI mechanisms, the limited therapies currently offered for HI treatment in the clinical setting stress the importance of discovering new targetable pathways. Efferocytosis is an immunoregulatory and homeostatic process of clearance of apoptotic cells (AC) and cellular debris, best described in the brain during neurodevelopment. The therapeutic potential of stimulating defective efferocytosis has been recognized in neurodegenerative diseases. In this review, we will explore the involvement of efferocytosis after a stroke and HI as a promising target for new HI therapies.

## 1. Introduction

Efferocytosis represents a physiologic process of phagocytic clearance of AC from the tissue by phagocytic cells promoting the resolution of inflammation [1]. Efferocytosis is carried out by professional phagocytes [2], such as microglia, macrophages, neutrophils or dendritic cells. Selected cells without the primary function of efferocytosis, such as oligodendrocytes are under certain conditions capable of performing efferocytosis, as well and are referred to as “non-professional” phagocytes [3,4]. Efferocytosis is a brain homeostatic process responsible for clearance of AC accumulated in tissue as a result of neurodevelopmental selection or various pathologies. Efferocytosis demonstrates neuroprotective effects through modulation of the local immunoregulatory mechanisms [5]. The timely and rapid containment of AC prevents their death by secondary necrosis that would lead to the release of cellular contents into the environment triggering inflammation [6]. In addition, ingestion of AC inhibits production of proinflammatory cytokines in efferocytes by autocrine and paracrine mechanisms [7], and reprograms efferocytes to release anti-inflammatory mediators [8,9], trophic factors [7,10] or bioactive lipids [11] contributing to the resolution of inflammation.

Efferocytosis is a pivotal process of error-free neurodevelopment, where clearing of unnecessary cells and synapses ensures normal redistribution of neural networks. Defective efferocytosis, both excessive or insufficient, contributes to the development of selected neurodevelopmental conditions [12,13,14]. For example, in Rett syndrome the principal mutation in methyl CpG binding protein 2 (Mecp2) [15] impacts microglial efferocytic functions, among others. The Mecp2-deficient microglia excessively engulf synapses weakened by the loss of Mecp2 expression leading to dismantling neural circuits [16]. The insufficient clearance of cellular debris stimulates the immune system and participates as a substrate for various neuropathologies (Table 1), including autoimmune diseases [17] or neurodegenerative processes, such as multiple sclerosis [18] or Alzheimer’s disease [19]. Escaping efferocytosis by overexpression of antiphagocytic signals, such as CD47 is a mechanism of brain cancer progression in glioblastoma [20]. Thus, well-functioning efferocytosis is a brain homeostatic and neuroprotective process that helps prevent inflammation [21,22] and contributes to inflammation resolution [23].

Could efferocytosis be harmful? While most of the studies describe efferocytosis as a neuroprotectant, some studies show possible detrimental effects. In the early phase of injury, a portion of the distressed penumbral neurons with potential for recovery reversibly expose phosphatidylserine, which is recognized by efferocytes as “eat me” signaling and prompts ingestion mediated by milk-fat globule EGF factor-8 (MFG-E8), thereby causing neuronal death [30]. While these neurons microscopically appear intact, it is possible that their apoptotic program is already turned on [31]. Similar observations are described during phagocytosis of lymphocytes that have undergone plasma membrane alterations of apoptosis without yet manifesting the nuclear condensation of apoptosis [32]. It is possible that this phase of efferocytosis is hard to precisely detect by current methods [33]. However, the inhibition of phagocytosis after transient brain ischemia prevents delayed neuronal loss and death of functional neurons [27]. Therefore, when evaluating mechanisms of efferocytosis, it is important to consider the limitations of detection of this pathway and consider the time points studied, especially in the rapidly developing neonatal brain where developmental changes in efferocytosis are not well-studied.

## 2. Regulation of Efferocytosis in Brain

### 2.1. Efferocytosis Signaling

Efferocytosis can be divided into four main phases that employ specific signaling. The first phase is initiated by “find me” signaling originating from AC that mediates recruitment of the efferocyte. The second “eat me” phase is triggered by signaling allowing the efferocyte to recognize the AC and initiate the “engulfment” phase of the AC, followed by “digestion” of the engulfed AC by the efferocyte [34].

Efferocytes have unique characteristics enabling them successfully to eliminate the AC from the tissue. The engulfment of the first AC triggers changes allowing engulfment of further cells in the process called continual efferocytosis [35,36]. The capacity of an efferocyte to ingest multiple cells subsequently [37,38], as well as the ability to digest a high load of nutrients from multiple ingested cells [39] ensures the necessary fast and efficient clearance of the AC from the injury site.

**Figure 1:**
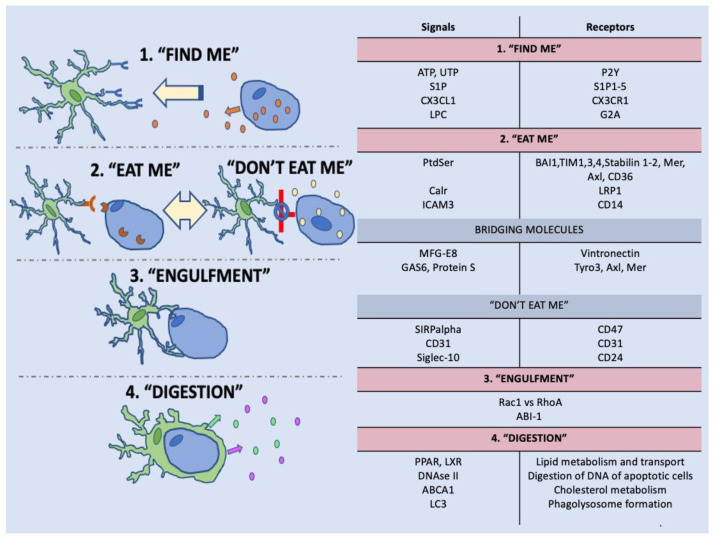
Four steps of efferocytosis: “Find me”, eat me”, “engulfment”, and “digestion” phase represent four main steps of efferocytosis characterized by a high level of complexity of their regulation that involves multiple signaling molecules, receptors, and pathways [30,34,43,45,48,52,53,54,55,56,57,58,59,60,61,62]. The engulfment of AC can promote the anti-inflammatory response of the efferocyte [63]. P2Y- purinergic P2Y receptor, S1P1-5- sphingosine 1-phosphate receptor subtype 1-5, CX3CR1- C-X3-C Motif Chemokine Receptor 1, G2A- G protein coupled receptor Gpr132, TIM- T cell immunoglobulin mucin receptor, Tyro3, Axl, Mer- TAM receptor tyrosine kinases, LRP1- LDL receptor related protein 1, ABI-1- Abl interactor 1, LC3- microtubule associated protein 1A/1B light chain 3, SIRPalpha- signal-regulatory protein-alpha, Siglec-10- sialic acid binding Ig-like lectin 10.

Efferocytes respond to multiple stimuli in the injured environment. Yet, efferocytes must recognize dead cells exhibiting “find me” signals that differ depending on the tissue and type of injury [40] (e.g., lysophosphatidylcholine—LPC, sphingosine-1-phosphate—S1P, CX3C motif chemokine ligand 1—CX3CL1, nucleotides adenosine triphosphate—ATP and uridine triphosphate—UTP) [41,42,43,44] and “eat me” signals (phosphatidylserine—PtdSer, calreticulin—Calr, intracellular adhesion molecule 3—ICAM3) [45,46,47,48] from distressed cells that release “help me” signals (interleukin-34, fibroblast growth factor 2, lipocalin-2) [49] and healthy cells with “don’t eat me” signals (CD31, CD47) [45,50]. Interestingly, some cells secrete “keep out signals”, for example, lactoferrin to selectively exclude certain efferocytes, such as eosinophils or neutrophils [45,51] underlying the cellular specificity of the efferocytosis process (Figure 1).

One of the best described “eat me” signals is phosphatidylserine, which is normally confined to the inner cytoplasmic leaflet of the plasma membrane by a “flippase”. Apoptosis activates a “scramblase” that quickly exposes phosphatidylserine on the cell surface [64] and stimulates a wide range of immunological responses, including the activation of anti-inflammatory and immunosuppressive pathways that prevent both local and systemic immune activation [65]. Other groups of signals capable of triggering efferocytosis in parallel with chemotactic signals are electrostatic signals resulting from damage of the cellular membrane [66]. In a model of endothelial efferocytosis, the endothelial AC were strongly negatively charged and attracted positively charged endothelial cells triggering reorganization of their cytoskeleton and sprouting [66]. The most abundant negatively charged structure in eukaryotic membranes is phosphatidylserine [65]. Phosphatidylserine is a glycerophospholipid with the ability to direct proteins with a positive charge [65,67]. The function of externalized phosphatidylserine as an “eat me” signal is complex and depends on the critical concentration or topology on the cell membrane [65]. A unique role in efferocytosis play selected soluble proteins (growth-arrest specific 6- Gas6, milk fat globule EGF factor 8- MFG-E8, protein S, C1q) that provide the link between the phagocyte and apoptotic cells by binding both to apoptotic signals on the AC, such as phosphatidylserine and the receptors on the phagocytes [45]. The engulfment phase is characterized by the activation of signaling pathways involved in cytoskeletal rearrangement. One of the fundamental regulators of formation and closure of the phagocytic cusp are Rho-family GTP-ases Rac Family Small GTPase 1 (Rac1) and Ras Homolog Family Member A (RhoA) that antagonistically regulate cytoskeletal rearrangement during efferocytosis [68]. Rac1 activates actin reorganization and facilitates the engulfment, whereas RhoA exhibits inhibitory functions [68]. Multiple signals and pathways are involved in Rac1 activation, including the Brain-specific angiogenesis inhibitor 1 (BAI-1) or integrinαvβ5 that couple with CrkII-Dock180-ELMO complex [69,70], the CED-1/MEGF10 or stabilin-2 via the CED-6/GULP adapter protein [60] or ABI-1 signaling pathway acting independently of the CED-10 Rac pathway or through CED-10 Rac [71]. The effective formation of the phagosome requires separation of the phagosome from the plasma membrane achieved by dynamin-actin crosstalk [72]. The phagosome undergoes further maturation leading to activation of signaling molecules and genes involved in degradation of its contents, such as regulators of lipid metabolism (ATP Binding Cassette Subfamily A Member 1- ABCA1, liver X receptor- LXR, peroxisome proliferator-activated receptor- PPAR) or nucleic acids (deoxyribonuclease II- DNAse II), etc. [34]. The efferocytosis signaling is a very complex but tightly regulated process to maintain homeostasis and health of the brain.

### 2.2. Cellular Specific Response

To perform flawless efferocytosis, efferocytes employ multiple receptors. Phosphatidylserine receptor, scavenger receptors, opsonin, complement, and pattern recognition receptors are all involved in the uptake of AC. These receptors are expressed in different densities on microglia, astrocytes, as well as neurons [73] resulting in efferocyte-AC specific responses. Specific AC activates AC-specific efferocytes. For example, in the model of autoimmune encephalitis, the apoptotic lymphocytes are ingested by microglia, oligodendrocytes, and astrocytes [32]. A specific AC activates gene expression signatures unique to each efferocyte, including metabolic and immunoregulatory genes [74] that influence the morphologic, metabolic, and inflammatory state of the efferocyte. This activation may happen by the exposure to AC only or can be prompted by the ingestion of the AC. Exposure of the efferocyte to AC induces SLC2A1-mediated glucose uptake resulting in a robust induction of an aerobic glycolysis program necessary for actin polymerization, continuous efferocytosis, and the establishment of an anti-inflammatory tissue environment via the SLC2A1-released lactate [8]. Efferocytes leverage AC metabolites, as AC engulfment elevates intracellular fatty acids concentration, which fuel mitochondrial respiration leading to anti-inflammatory reprogramming and IL-10 secretion [63].

### 2.3. Tissue Specific Response

An additional level of complexity to the regulation of efferocytosis is highlighted by a phagocyte-tissue specific response as represented by the response of phagocytes to phosphatidylserine. The phosphatidylserine receptor, T-cell immunoglobulin-, and mucin-domain-containing molecule (Tim4) is required for the efficient efferocytosis by resident peritoneal macrophages, Kupffer cells, and CD169^+^ skin macrophages, whereas thioglycollate-elicited peritoneal macrophages and cultured microglial efferocytosis are independent of Tim4 [62]. In vivo, however, while Tim4-lacking microglia are still able to recognize the AC, they exhibit distinct clearance defects. Specifically, Tim4 is required for phagosome stabilization and brain-specific angiogenesis inhibitor 1 (BAI1) controls the formation of phagosomes around dying neurons and cargo transport [75]. Tim4, together with BAI1 are crucial for efficient microglial performance of efferocytosis in vivo underlying the importance of comparing the in vivo vs. in vitro cell culture studies and considering the tissue-specificity of efferocytosis. The spatiotemporal location in the brain defines the regulatory pathways of efferocytosis, as well. In neurogenic regions of the adult mice brain, microglial efferocytic functions depend on TAM receptor tyrosine kinases Mer and Axl and both TAM receptor ligands, growth arrest specific gene 6 (Gas6) and protein S signaling [76]. Gas6 stimulates phagocytosis by bridging the phosphatidylserine residues on the surface of AC to the Axl/Mer [77]. The deficiency in microglial Mer and Axl leads to the marked accumulation of AC specifically in neurogenic regions of the CNS [76]. Similarly to phosphatidyserine, the Gas6 has a dual effect of stimulation of efferocytosis, as well as on limiting inflammation as the Gas6 also suppress lipopolysaccharide-induced expression of the inflammatory molecules IL-1β and iNOS expression through suppression of promoter activity [77]. The understanding of the whole orchestra of interactions necessary for the process of efferocytosis is just beginning, but the regulatory pathways of efferocytosis seem to be tissue, AC, event, and phagocyte-specific. The timing after injury and developmental stage likely play a role, as well. This may be true especially for the neonatal brain, as profound differences have been described in response to injury such as brain HI [78,79].

### 2.4. Brain Efferocytosis Signals: IL-4-STAT6-PPARγ-Arginase-1 Pathway

The interleukin-4 (IL-4)-signal transducer and activator of transcription 6 (STAT6)-peroxisome proliferator-activated receptor-γ (PPARγ)-arginase-1 (Arg1) signaling axis is one of the key pathways regulating microglial/macrophage efferocytosis in the HI brain. IL-4 is an upstream regulator of PPARγ via STAT6. STAT6 amplifies the PPARγ activity via binding to the enhancer of PPARγ target genes [80].

In the post-ischemic brain, the nuclear receptor PPARγ and STAT6 are the only two upstream regulators predicted to be strongly activated in brain macrophages. The downstream targets of these two regulators include anti-inflammatory factors and growth factors (e.g., IL-10, Arg 1, IGF1, LIF, GDF-15, FGF1), nuclear receptors (e.g., LXR-α, NR4A1), and efferocytic receptors and transporters (e.g., CD36, ABCA1) [81]. PPARγ is a ligand-dependent transcription factor that regulates the expression of specific target genes [82], including genes involved in regulating efferocytosis. PPARγ is induced during annexin 1-mediated microglial efferocytosis of apoptotic neurons [83], and also regulates CD36-mediated phagocytosis in the hemorrhagic stroke model [84]. In addition, PPARγ regulates inflammation by interaction with other transcriptional factors and signaling proteins, such as NFκB, by transrepression of MAP kinases or by repressing the transcription of inflammatory mediator genes [82]. In focal cerebral ischemia, PPARγ inhibits COX-2 and decreases expression of iNOS and IL-1β [85]. Similarly to PPARγ, STAT6 also regulates efferocytosis of neurons and inflammatory gene signature in microglia/macrophages. STAT6 deficiency results in an enlarged infarct volume and worse neurological outcomes early after an experimental stroke [23].

**Figure 2:**
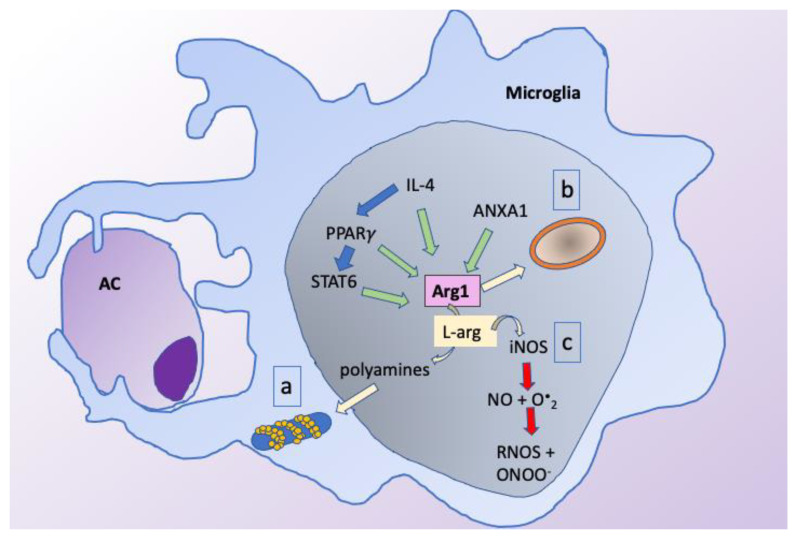
Different roles of Arg1 in efferocytosis: Arg1 is a downstream target of multiple regulators of efferocytosis. Arg1 is involved in engulfment by regulation of phagolysosome formation (**a**) in lysosomal digestion (**b**) and exhibits anti-inflammatory effects by reducing NO radical formation (**c**).

Arg1 is a downstream target of PPARγ [83,86] and STAT6 [87]. Additional regulatory molecules of efferocytosis, such as annexin-A1 [88] and IL-4 [87], have been shown in independent studies to be associated with increased Arg1 expression. Arg1 synthesis at the injury site is stimulated by the L-arginine released from AC [35] or mechanically by elongation of macrophages [89] and is associated with the macrophage switch towards the anti-inflammatory phenotype [89,90]. Arg1 exhibits multiple effects on efferocytosis (Figure 2). During the engulfment phase, Arg1 impacts efferocytosis mechanistically by regulation of the phagolysosome formation. The key cell biological process in AC internalization is actin remodeling around the forming phagosome [91]. Arg1 metabolizes L-arginine released from AC to polyamines and promotes Rac1 activation resulting in actin polymerization for formation of cytoskeleton of the phagosome [35]. In addition, Arg1 is vital for continuous efferocytosis. While Arg1 deletion has no effect on the ingestion of the first AC during efferocytosis, it inhibits the ingestion of the subsequent AC, thus impacting the performance of the continuous efferocytosis [35]. Arg1 regulates the digestion phase of efferocyte, as well. The lack of Arg1 leads to the reduction of Ragulator-Rag complex, an essential regulator of microglial lysosomal activity [92], resulting in impairment of microglial digestion [93]. Furthermore, Arg1 contributes to the control of inflammation achieved by suppression of nitric oxide (NO) and superoxide production [94], etc. These findings suggest the pivotal role for Arg1 in microglial efferocytosis, underlined by findings of decreased phagocytosis in rat microglial cells after Arg1 repression by siRNA [93]. Arg1 is primarily abundant in activated microglia and macrophages during early stages of neurodevelopment and after neonatal HI in comparison to the adult brain [95]. Therefore, we can speculate that the process of Arg1 related efferocytosis is highly developmentally-dependent and significantly impacts neuroinflammation and neuroregeneration after HI injury.

## 3. Brain Efferocytes: Microglia and Beyond

### 3.1. Brain Efferocytes

The major professional phagocytes performing efferocytosis in the brain under normal conditions and in disease are resident microglia [96]. Peripheral blood macrophages are involved in efferocytosis upon the insult [81], but it is unclear whether they participate in the maintenance of brain homeostasis also under normal conditions. The “non-professional” efferocytes include oligodendrocytes [4,32,97], astrocytes [98], endothelial cells [99,100], pericytes [101,102] in the brain or Schwann cells in the peripheral nervous system [103], etc. The “non-professional” efferocytes substantially contribute to brain efferocytosis, as well. Astrocytes eliminate synapses either by direct engulfment or indirectly. The direct engulfment utilizes the MERTK and MGEF10 phagocytic pathways that both recognize the “eat me” signals such as phosphatidylserine residues on the AC [104,105]. The indirect engulfment is carried out by TGF-β secreting astrocytes. TGF-β induces the C1q expression in retinal ganglion cells leading to opsonization of the unwanted synapses that are subsequently eliminated by complement-mediated microglial phagocytosis [106,107]. In addition to synaptic clearance, astrocytes participate in engulfment of the neuronal AC, as well. In cerebellum, astrocytes engulfed the apoptotic neurons via the MEGF10 pathway [108]. Astrocytes even in a healthy CNS express TAM phagocytic receptors and contribute to the clearance of cellular debris especially under circumstances of impaired microglial function [109]. The ability of oligodendrocytes to participate in phagocytosis in the cell culture has been also described [110]. A unique group of efferocytes is formed by doublecortin (DCX)-positive neuronal progenitor cells found within the neurogenic zones during adult neurogenesis. These cells require intracellular engulfment protein ELMO1 for their function and ELMO1 deficiency reduces the uptake by DCX^+^ cells resulting in accumulation of apoptotic nuclei in the neurogenic niches and impaired neurogenesis, suggesting that proper phagocytic functions of DCX^+^ cells significantly contribute to adult neurogenesis [111]. It is likely that more cells in the brain capable of efferocytosis are yet to be discovered and that a distinct group of efferocytes is activated during the neurodevelopmental pruning [112] and routine “maintenance” of the brain compared to pathological processes. This is due to the fact that brain efferocytes differ in their ability to execute particular steps of efferocytosis, such as sensing cells, speed of engulfment, digestive capacity, and secretion of trophic anti-inflammatory factors, etc.

### 3.2. Differences between Professional and Non-Professional Efferocytes

Professional efferocytes differ in their ability to execute efferocytosis from non-professional phagocytes. For example, in vivo astrocytes and microglia rapidly polarize their processes towards dying cells within 2 to 3 h after laser induction of apoptosis. However, microglia take precedence in engulfing the dying cells [113]. The observed onset of efferocytosis is model dependent, as in an in vivo model of focal cerebral ischemia, the phagocytosis of neurons does not occur until at least 24 h after focal cerebral ischemia [27]. The professional efferocytes regulate efferocytic functions of the nonprofessional efferocytes. For example, macrophages by releasing insulin-like growth factor-1 and microvesicles redirect the phagocytosis and the type of material engulfed by non-professional efferocytes, specifically limiting the uptake of larger AC [114]. Time-lapse recordings of cells in culture show that professional efferocytes ingest AC faster, within minutes than non-professional efferocytes, where ingestion takes a few hours [31]. While non-professional efferocytes recognize the AC quickly, they delay its ingestion [31,115]. This different timing of ingestion has an important impact on the process of efferocytosis. AC have to appear late in the death process before they can stimulate non-professional efferocytes leaving this process to professional efferocytes [31] which are much more efficient. The digestion of professional efferocytes is also faster, lasting only 1–2 min, compared to hours in non-professional efferocytes [31]. The whole process of microglial efferocytosis in zebrafish takes approximately 20 min with phagosome formation within 8 min [75]. These observed differences between the non-professional and professional efferocytes suggest higher phagocytic efficiency of professional efferocytes. However, the non-professional efferocytes are vital in the process of efferocytosis. While the phagocytic efficiency of astrocytes remains lower compared to microglia, astrocytes support efferocytosis later in the injury when the microglial density decreases [113]. Other glial cells, such as NG2 glia also respond to dying cells by rapid polarization of their processes towards the cell corpse. However, only microglia migrate to fully engulf the dying cell and proximal dendrites. The NG2 glial response is observed at 6 h after exposure and polarization remains at 24 h despite complete removal of the dead cells [113]. NG2 glia fill the space left following the corpse removal [113], where they may play a role in neuroregeneration, as NG2 glial cells participate in the remyelination and are capable of conversion to neurons and astrocytes [116,117].

### 3.3. Differences among Professional Efferocytes

The differences in performance of efferocytosis exist also among brain professional phagocytes, macrophages, and microglia. In vitro, macrophages respond much faster than microglia when encountering apoptotic neurons resulting in earlier onset and plateau of efferocytosis [23]. Furthermore, macrophages manifest higher phagocytic capacity compared to microglia [118]. However, microglia are vital in the long-term recovery and repair process, as 7 days after a stroke in vivo resident microglia again become the predominant phagocyte, while monocytes diminish in ischemic brain [118]. A different response is seen in the model of spinal cord injury, where microglia are involved in the early response to injury by phagocytosing damaged and degenerating tissue. Later, macrophages of peripheral origin predominantly contribute to phagocytosis but are less efficient at processing CNS debris. Microglia in this model also remain viable longer while macrophages die and in situ may contribute to secondary damage [119]. The capacity to clear the AC from the injury site also differs among professional phagocyte subsets. The anti-inflammatory macrophages show a 1.5- to 2-fold higher capacity for both binding and uptake of apoptotic cells compared with dendritic cells and the proinflammatory macrophage phenotype [120]. These fundamental differences in efferocytosis observed based on the tissue, cells, and the trigger suggest that brain efferocytosis is a unique process, where professional and non-professional phagocytes are of equal importance in order to maintain homeostasis.

## 4. Efferocytosis during Neurodevelopment and after a Stroke

### 4.1. Neurodevelopmental Aspects of Efferocytosis

Brain efferocytosis is a robust homeostatic process occurring during neurodevelopment that serves to remove unnecessary and excessive neurons [121] and synapses to ensure proper developmental patterning of the structure and function of the brain [122]. The selected cellular and synaptic clearance continues to adulthood as part of the regulation of neurogenesis [123]. Ongoing efferocytosis in the adult brain determines the final number of newly formed neuroblasts that eventually incorporate into circuitry as observed in the hippocampus [123]. Microglia are known for their fundamental role in regulation of the size of the neural precursor cell pool in the developing cerebral cortex [124] and for synaptic pruning in a developing brain [125]. While the focus has been mostly directed on the microglial role in fine-tuning the brain structure during development, the contribution of non-professional phagocytes to developmental efferocytosis is equally important and cannot be overlooked. Astrocytes are an example of efferocytes vital for synaptic pruning and remodeling [104]. The stage of developmental maturity of the brain likely guides the phagocytic capacity of the efferocytes and impacts the response to a stroke. Efferocytosis is vigorous in the neonatal brain and declines with age [126]. Some of the enzymes involved in efferocytosis, such as Arg1 undergo developmental changes with decreasing levels during neurodevelopment [95]. The developmental efferocytic response to a stroke based on different ages still needs to be understood.

### 4.2. Efferocytosis after an Ischemic Stroke

The ischemic environment after a stroke triggers the genetic reprogramming of efferocytes resulting in their morphological changes, proliferation, and polarization [127]. Microglia and macrophages then differentiate into various phenotypes with specific spatiotemporal patterns at the injury site [128]. The ischemia-induced genomic reprogramming enhances the efferocytic capacity of phagocytes resulting in the upregulation of plasma membrane receptors and extracellular bridge molecules necessary for recognition of AC, a cytoskeletal rearrangement enabling the engulfment of AC and phagosome internalization and activation of transcription regulators that engage in the production of anti-inflammatory and trophic factors [81]. In addition to stimulatory effects of the injured tissue on efferocytosis, the injured tissue may have the opposite effect and cause microglial dysfunction leading to impaired efferocytosis. A common associated finding of brain HI is glutamate toxicity that leads to neuronal hyperactivity. The widespread ATP release during neuronal hyperactivity blinds microglia to the ATP microgradients released by apoptotic cells as “find me” signals with subsequent accumulation of AC [129]. Thus, the phagocyte-AC-tissue represents a dynamic unit of complex interactions with impact on the neuroprotection that is still not understood.

**Figure 3:**
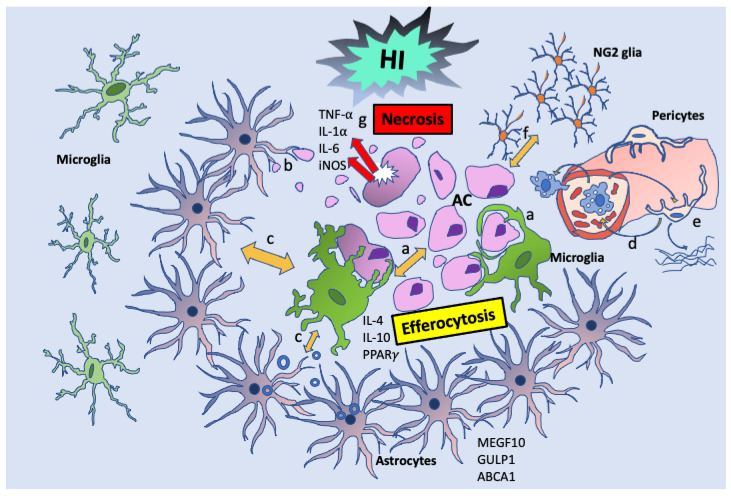
Efferocytosis crosstalk in HI: The AC reprograms efferocytes to express specific gene signatures that regulate the morphologic, metabolic, and inflammatory state of efferocytes. Microglia accumulate at the injury site, where they touch, engulf, and enwrap the AC (**a**). Astrocytes locate to the penumbra and participate in phagocytosis of synaptic debris (**b**) [122]. Astrocytes and microglia reciprocally inhibit or stimulate their efferocytic function and anti-inflammatory phenotype (**c**) [123,124,125]. Pericytes recruit macrophages and enhance their phagocytic activity (**d**) and macrophages stimulate pericytes to produce extracellular matrix proteins (**e**) [98]. NG2 glia fill-in the empty space following AC removal and support neuroregeneration by remyelination and/or conversion to neurons or astrocytes (**f**) [111,112]. The defective efferocytosis leads to secondary necrosis and inflammation (**g**), while well-functioning efferocytosis promotes neuroregeneration.

After a stroke, efferocytes undergo specific spatiotemporal changes at the injury site. Within 24–48 h after injury, a group of microglia form a barrier and surround the lesion possibly to prevent expansion. The protective phenotype expressing Ym1 and CD206, etc., populate the ischemic core [128]. Microglia are faster to enter the core of the injury site, within 24 h, while the motility of astrocytes is slower. Astrocytes locate to the penumbra of the injury site during the later phase of the injury, starting at 3 days post the ischemic insult. The penumbral astrocytes express the ABCA1 pathway molecules, MEGF10 and GULP1 and participate in phagocytosis of synaptic debris [130]. Astrocytes engulf diffuse apoptotic bodies derived from the dendritic arbors of dying neurons, by polarizing their distal processes without exhibiting cell body migration. Microglia predominantly phagocytose dendrites, cell bodies, and nuclei by migrating towards these structures and completely engulfing them [113]. At 7 days, phagocytosis of neurons and debris removal are prevalent [128]. The coordinated responses by microglia and astrocytes in clearance of neuronal corpses are tightly regulated by the receptor tyrosine kinase *Mertk* [113], but as for the astrocyte-microglial crosstalk (Figure 3) in brain hypoxic-ischemic injury, we are only beginning to understand. Astrocytes can inhibit the efferocytic function of microglia [131] or stimulate microglial anti-inflammatory phenotype by releasing exosomes containing miR-873a-5p [132], while microglial downregulation of P2y1 receptor leads to transformation of astrocytes to neuroprotective phenotype [133]. This communication among cells after a stroke is not limited to microglia-astrocytes only. Pericytes indirectly recruit macrophages to the injury site and promote phagocytic activity in macrophages. The macrophages process myelin debris and produce trophic factors, enhancing PDGFRβ signaling in pericytes leading to the production of extracellular matrix proteins and oligodendrogenesis [102]. Thus, the process of efferocytosis of dying neurons after a stroke is characterized by active communication among multiple cells taking over specific functions.

## 5. Efferocytosis as a Therapeutic Modality in Stroke

### 5.1. Timing Approach to Treatment

Targeting the efferocytosis pathway represents a new promising strategy for stroke therapies. A beneficial approach might be to enhance as well as inhibit efferocytosis. The effects of efferocytosis-targeted therapies for overall post stroke recovery will depend on the timing of their administration after the injury. Early phases of ischemic injury are characterized by infiltration of the reparative microglial/macrophage phenotype that declines over time, and are replaced by the proinflammatory, potentially harmful phenotypes [134]. Therapies aimed at supporting the polarization of efferocyte to the anti-inflammatory phenotype may suppress inflammation and improve outcomes. In early phases, it is important to limit the ingestion of distressed yet viable neurons which could lead to improved neuronal survival. At later time points when efferocytosis is decreased, therapies that stimulate the process of efferocytosis could enhance post stroke recovery [134].

### 5.2. Selected Therapies and Translational Potential

Multiple molecules have been successfully studied and selected molecules are already used as FDA-approved pharmaceuticals for other indications. Administration of PPARγ agonists troglitazone or pioglitazone 24 h before and at the time of cerebral infarction dramatically reduced infarction volume and improved neurological function following middle cerebral artery occlusion in rats [85]. The beneficial effects are observed also later after the injury at 22 days [85]. Importantly, rosiglitazone has been shown to improve outcomes in patients with Alzheimer’s disease [135] and adult stroke [136] supporting the translational potential of efferocytosis-targeted therapies.

Utilizing therapies that mimic products released at the injury site during efferocytosis carries a therapeutic potential, as well. For example, administration of IL-4, a cytokine released after an injury from challenged neurons enhances microglial phagocytosis of AC supporting brain cleanup after an ischemic stroke that translates to better outcomes and decreased lesion volume [137].

A fundamental role to prevent neuroinflammation after a stroke is clearance of myelin debris. Myelin debris is toxic for brain cells as it inhibits remyelination critical for angiogenesis and neurogenesis in post-stroke recovery [138]. Effective clearance of inhibitory myelin debris stimulates neurotrophin synthesis, and formation of blood vessels in a model of spinal cord injury [139]. Restoration of myelin debris phagocytosis can be enhanced by the administration of retinoid X receptor (RXR) agonists [140], PPARγ agonists [141] or the E6020, a synthetic TLR4 agonist [142]. E6020 accelerated myelin debris clearance results in Schwann cell infiltration and remyelination in rat spinal cord [142].

The undesired effects of excessive efferocytosis of distressed yet viable neurons can be prevented by therapies focused on administration of extracellular vesicles containing molecules such as miR-98. In a model of ischemic stroke, miR-98-loaded extracellular vesicles prevented the stressed but viable neurons from microglial phagocytosis [143]. The miR-98 acts via the platelet activating factor receptor (PAFR) that mediates efferocytosis as observed during engulfment of excitatory synapses of hippocampus in a model of experimental auto-immune encephalomyelitis [144] or apoptotic thymocytes in cell culture [145].

Efferocytosis has a vital role in the treatment of the hemorrhagic stroke, as well. While ischemic and hemorrhagic stroke are different pathologies, they share similarities in some of the activated efferocytic signals after injury. For example, the IL-4/STAT6 pathway is one of the canonical pathways of hemorrhage clearance in the brain [146] and STAT6 signaling is activated also after the ischemic stroke [23]. PPARγ agonists, showing neuroprotective effects in an ischemic stroke, help in hemorrhagic stroke by hematoma absorption by 2.3-fold, resulting in decreased neuronal damage, and improved functional recovery [147]. Stimulation of phagocytosis to enhance the clearance of the hematoma is a desirable effect also of the Nrf2 activator, sulforaphane. One possible mechanism is induction of CD36 expression and stimulation of phagocytosis [148]. An FDA-approved selective RXR agonist, bexarotene shows effectiveness in both ischemic and hemorrhagic stroke [149]. Treatment of intracerebral hemorrhagic stroke by bexacarotene leads to increased expression of key receptors responsible for erythrophagocytosis by macrophages after intracerebral hemorrhage, including Axl and CD36. The bexarotene treatment enhances erythrophagocytosis and improves neurological outcomes [150].

While efferocytosis after a neonatal brain stroke is a specific process, the overlap in the activation of certain pathways in different brain pathologies and their successful pharmacological targeting could inspire efferocytosis-targeted therapies used in a neonatal stroke.

## 6. Conclusions

Efferocytosis is a known HI pathway but underexplored in the neonatal brain. While it is a pivotal mechanism of clearance of the unnecessary cells and synapses during neurodevelopment, current studies suggest the possible role in neuroprotection. Future studies should focus on exploring the differences in execution of efferocytosis related to neurodevelopment to better understand and define the changes based on the age and timing after an injury. In addition to improving our understanding of the HI pathophysiology, we believe efferocytosis could serve as a source for new therapies for neonatal HI.

## Figures and Tables

**Table 1:** Defective efferocytosis-based brain pathologies.

Pathological Condition	Observed Defects in Efferocytosis
Glioblastoma [24]	Overexpression of “don’t eat me” CD47 leading to efferocytosis escape
Alzheimer’s disease [19,25]	Accumulation of the plaque Loss of TREM2 reduces phagocytic clearance of the plaque
Rett syndrome [16]	Excessive engulfment of synapses
Down syndrome [26]	Overactive microglia with altered neuronal dendritic-spine turnover in hippocampus
Neurodegeneration [27]	Failure to recognize distressed but viable neurons after stroke
Parkinson‘s disease [28]	C3-complement system-induced phagocytosis of dopaminergic neurons Ineffective α-syn clearance from degenerating neurons
Multiple sclerosis [29]	Internalizing the intact myelin; insufficient clearance of damaged myelin

## Data Availability

Data sharing not applicable.
